# Crystal structure of *catena*-poly[[silver(I)-{μ-2,6-bis­[(1*H*-pyrazol-1-yl)meth­yl]pyridine-κ^3^ *N* ^1^,*N* ^2^:*N* ^2′^}] nitrate]

**DOI:** 10.1107/S2056989015004120

**Published:** 2015-03-07

**Authors:** Daeyoung Kim, Sung Kwon Kang

**Affiliations:** a Department of Chemistry, Chungnam National University, Daejeon 305-764, Republic of Korea

**Keywords:** crystal structure, silver(I) complex, one-dimensional coordination polymer, 2,6-bis­[(1*H*-pyrazol-1-yl)meth­yl]pyridine

## Abstract

In the title complex, {[Ag(C_13_H_13_N_5_)]NO_3_}_*n*_, the Ag^I^ atom is coordinated by three N atoms from two bidentate/monodentate pyrazolylpyridyl ligands to form a distorted trigonal–planar geometry [range of angles: 83.34 (6) (chelate ring) to 139.15 (7) °]. The chelate ring has a distorted boat conformation. The dihedral angle between the pyridyl ring and the coordinating pyrazolyl ring is 67.22 (6)°. The non-coordinating pyrazolyl ring is twisted by 62.97 (7)° from the pyridyl ring. In the crystal, the complex cations are arranged in polymeric chains along the *c*-axis direction, with the nitrate counter-anions situated in between. Weak C—H⋯O hydrogen bonds link the ions into a three-dimensional network.

## Related literature   

For related metal complexes, see: Reger *et al.* (2005 ▸); Sharma *et al.* (2011 ▸); Hurtado *et al.* (2011 ▸). For the synthesis of 2,6-bis­[(1*H*-pyrazol-1-yl)meth­yl]pyridine, see: Singh *et al.* (2003 ▸); Son *et al.* (2014 ▸); Watson *et al.* (1987 ▸).
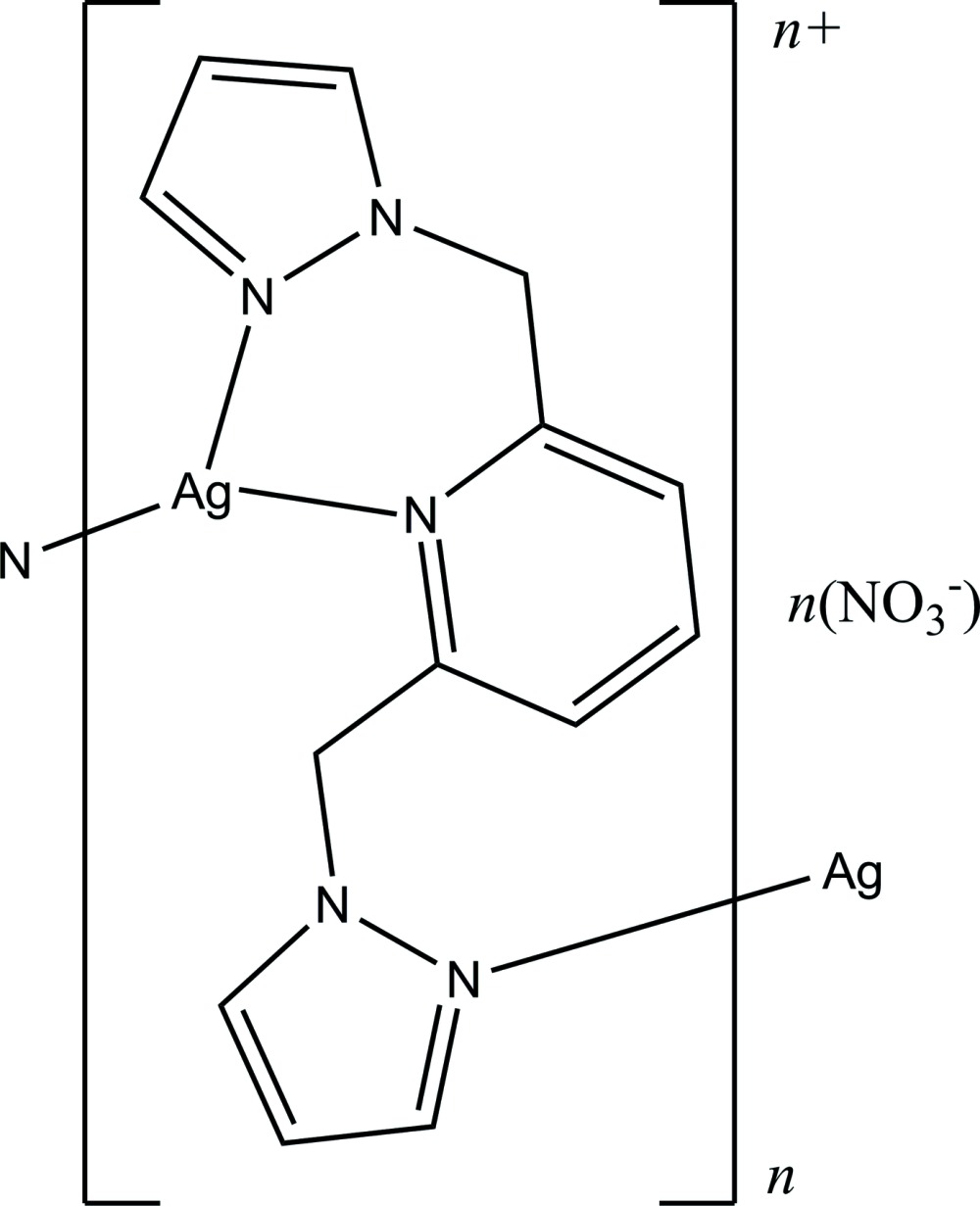


## Experimental   

### Crystal data  

[Ag(C_13_H_13_N_5_)]NO_3_
*M* *_r_* = 409.16
Monoclinic, 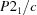

*a* = 9.9604 (6) Å
*b* = 14.3192 (9) Å
*c* = 10.6878 (7) Å
β = 98.9100 (9)°
*V* = 1505.95 (16) Å^3^
*Z* = 4
Mo *K*α radiation
μ = 1.36 mm^−1^
*T* = 296 K
0.25 × 0.23 × 0.21 mm

### Data collection  

Bruker SMART CCD area-detector diffractometer
Absorption correction: multi-scan (*SADABS*; Bruker, 2002 ▸) *T*_min_ = 0.546, *T*_max_ = 0.726
12683 measured reflections
3618 independent reflections
3087 reflections with *I* > 2σ(*I*)
*R*_int_ = 0.017

### Refinement  

*R*[*F* ^2^ > 2σ(*F* ^2^)] = 0.027
*wR*(*F* ^2^) = 0.063
*S* = 1.04
3618 reflections
208 parameters
H-atom parameters constrained
Δρ_max_ = 0.43 e Å^−3^
Δρ_min_ = −0.39 e Å^−3^

### 

Data collection: *SMART* (Bruker, 2002 ▸); cell refinement: *SAINT* (Bruker, 2002 ▸); data reduction: *SAINT*; program(s) used to solve structure: *SHELXS2013* (Sheldrick, 2008 ▸); program(s) used to refine structure: *SHELXL2013* (Sheldrick, 2015 ▸); molecular graphics: *ORTEP-3 for Windows* (Farrugia, 2012 ▸); software used to prepare material for publication: *WinGX* (Farrugia, 2012 ▸).

## Supplementary Material

Crystal structure: contains datablock(s) global, I. DOI: 10.1107/S2056989015004120/zq2231sup1.cif

Structure factors: contains datablock(s) I. DOI: 10.1107/S2056989015004120/zq2231Isup2.hkl

**Fig. 1.**
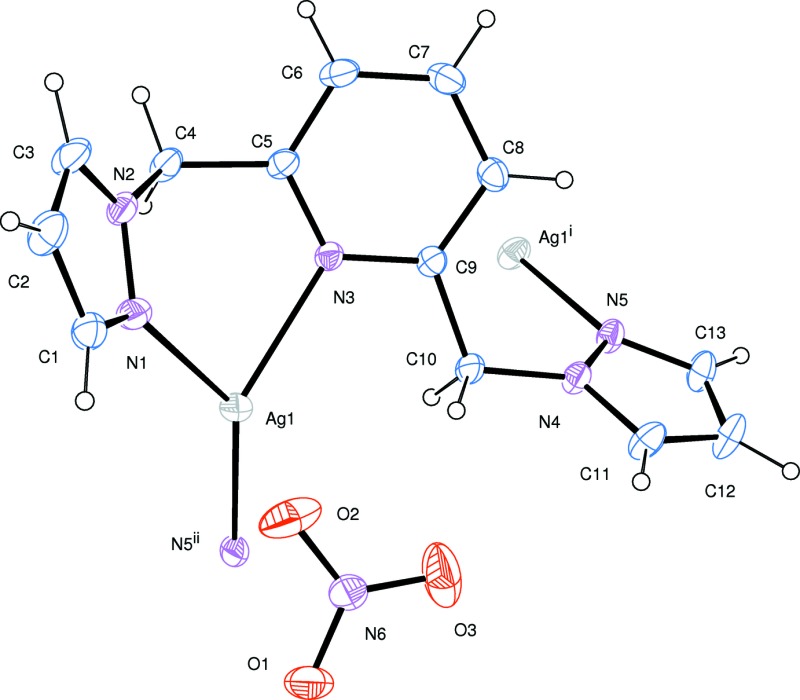
Structure of the title compound, showing the atom-numbering scheme and 30% probability ellipsoids [symmetry codes: (i) x, -y + 1/2, z + 1/2; (ii) x, -y + 1/2, z - 1/2].

Click here for additional data file.x y z x y z . DOI: 10.1107/S2056989015004120/zq2231fig1.tif
Structure of the title compound, showing the atom-numbering scheme and 30% probability ellipsoids [symmetry codes: (i) *x*, −*y* + 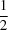, *z* + 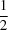; (ii) *x*, −*y* + 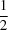, *z* − 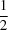].

**Fig. 2.**
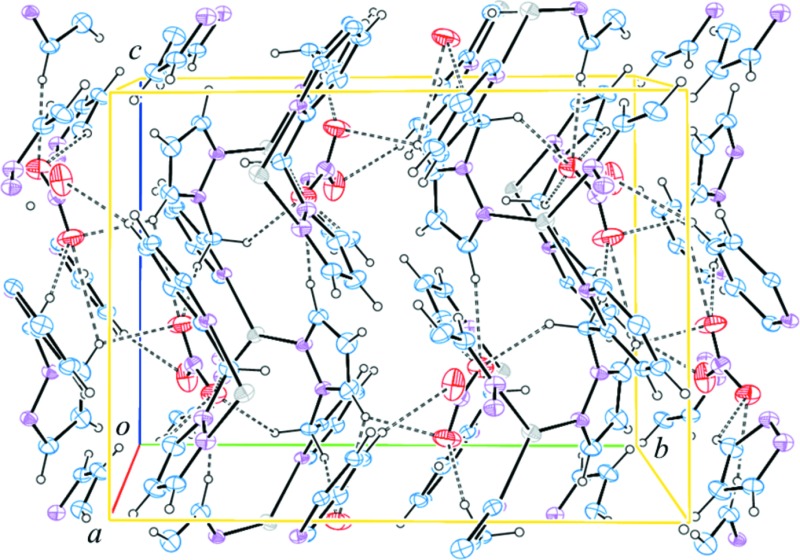
Part of the crystal structure of the title complex, showing the 3-D network of molecules linked by weak C—H···O hydrogen bonds (dashed lines).

. DOI: 10.1107/S2056989015004120/zq2231fig2.tif
Part of the crystal structure of the title complex, showing the 3-D network of mol­ecules linked by weak C—H⋯O hydrogen bonds (dashed lines).

CCDC reference: 1051419

Additional supporting information:  crystallographic information; 3D view; checkCIF report

## Figures and Tables

**Table 1:** Hydrogen-bond geometry (, )

*D*H*A*	*D*H	H*A*	*D* *A*	*D*H*A*
C3H3O1^i^	0.93	2.49	3.206(3)	134
C4H4*A*O1^i^	0.97	2.48	3.347(3)	149
C10H10*A*O3	0.97	2.38	3.277(3)	154
C11H11O3^ii^	0.93	2.43	3.195(3)	140
C13H13O1^iii^	0.93	2.44	3.355(3)	167

Symmetry codes: (i) 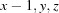; (ii) 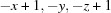; (iii) 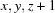.

## supplementary crystallographic information

### S1. Structural commentary

The metal complex with the tridentate ligand 2,6-bis­((1*H*-pyrazol-1-yl)methyl)­pyridine is reported as a catalyst of polyethyl­ene polymerization (Hurtado *et al.*, 2011; Watson *et al.*, 1987). As a contribution to this field, we report herein the crystal structure of the title compound. In the cation part of the title compound, two ligands are linked by one silver atom. One pyrazolyl ring and the central pyridyl ring are coordinated to a second silver atom, as pictured in Fig 1. This bridging structure has shown that silver(I) complex can produce sophisticated coordination architectures and supra­molecular arrays. The Ag—N distances are within the range of 2.165 (2) - 2.313 (2) Å with the silver atom in an almost trigonal planar arrangement and the sum of N—Ag—N angles being 359.5 ° with the range of 83.34 (6) - 139.15 (7) °. The crystal structure features *π-π* stacking inter­actions between pyridyl rings [centroid-centroid distance = 3.700 (3) Å] and geometric constraints imposed by coordination of the ligand to the silver atoms, as pictured in Fig 2.

### S2. Synthesis and crystallization

To a stirred solution of Ag(NO_3_) (0.051g, 0.3 mmol) in aceto­nitrile (5 ml) was added a solution of 2,6-bis­((1*H*-pyrazol-1-yl)methyl)­pyridine (0.072 g, 0.3 mmol) in aceto­nitrile (5 ml) at room temperature. After 24 h of stirring, a white powder was formed. The product was washed with di­ethyl ether. Single crystals of the title complex were obtained from its aceto­nitrile solution by slow evaporation of the solvent at room temperature within 2 weeks.

### S3. Refinement

All H atoms were positioned geometrically and refined using a riding model, with C—H = 0.93 - 0.98 Å, and with U_iso_(H) = 1.2U_eq_(C).

### Crystal data

**Table:**

[Ag(C_13_H_13_N_5_)]NO_3_	*F*(000) = 816
*M**_r_* = 409.16	*D*_x_ = 1.805 Mg m^−^^3^
Monoclinic, *P*2_1_/*c*	Mo *K*α radiation, λ = 0.71073 Å
Hall symbol: -P 2ybc	Cell parameters from 6606 reflections
*a* = 9.9604 (6) Å	θ = 2.4–28.3°
*b* = 14.3192 (9) Å	µ = 1.36 mm^−^^1^
*c* = 10.6878 (7) Å	*T* = 296 K
β = 98.9100 (9)°	Block, colourless
*V* = 1505.95 (16) Å^3^	0.25 × 0.23 × 0.21 mm
*Z* = 4	

### Data collection

**Table:**

Bruker SMART CCD area-detector diffractometer	3087 reflections with *I* > 2σ(*I*)
Radiation source: fine-focus sealed tube	*R*_int_ = 0.017
φ and ω scans	θ_max_ = 28.3°, θ_min_ = 2.1°
Absorption correction: multi-scan (*SADABS*; Bruker, 2002)	*h* = −13→13
*T*_min_ = 0.546, *T*_max_ = 0.726	*k* = −18→19
12683 measured reflections	*l* = −14→10
3618 independent reflections	

### Refinement

**Table:**

Refinement on *F*^2^	0 restraints
Least-squares matrix: full	Hydrogen site location: inferred from neighbouring sites
*R*[*F*^2^ > 2σ(*F*^2^)] = 0.027	H-atom parameters constrained
*wR*(*F*^2^) = 0.063	*w* = 1/[σ^2^(*F*_o_^2^) + (0.0274*P*)^2^ + 0.685*P*] where *P* = (*F*_o_^2^ + 2*F*_c_^2^)/3
*S* = 1.04	(Δ/σ)_max_ = 0.001
3618 reflections	Δρ_max_ = 0.43 e Å^−^^3^
208 parameters	Δρ_min_ = −0.39 e Å^−^^3^

### Special details

**Table:**

Geometry. All e.s.d.'s (except the e.s.d. in the dihedral angle between two l.s. planes) are estimated using the full covariance matrix. The cell e.s.d.'s are taken into account individually in the estimation of e.s.d.'s in distances, angles and torsion angles; correlations between e.s.d.'s in cell parameters are only used when they are defined by crystal symmetry. An approximate (isotropic) treatment of cell e.s.d.'s is used for estimating e.s.d.'s involving l.s. planes.

### Fractional atomic coordinates and isotropic or equivalent isotropic displacement parameters (Å^2^)

**Table:**

	*x*	*y*	*z*	*U*_iso_*/*U*_eq_	
Ag1	0.11928 (2)	0.23756 (2)	0.31490 (2)	0.04028 (7)	
N1	−0.04629 (18)	0.16608 (14)	0.17705 (18)	0.0399 (4)	
N2	−0.17162 (18)	0.15774 (13)	0.20861 (17)	0.0370 (4)	
N3	0.00565 (16)	0.17147 (12)	0.46594 (15)	0.0297 (4)	
N4	0.30868 (17)	0.12786 (13)	0.70046 (17)	0.0341 (4)	
N5	0.28527 (18)	0.16529 (13)	0.81157 (16)	0.0358 (4)	
C1	−0.0480 (2)	0.11349 (17)	0.0749 (2)	0.0433 (5)	
H1	0.0253	0.106	0.0314	0.052*	
C2	−0.1733 (3)	0.07115 (19)	0.0413 (3)	0.0516 (6)	
H2	−0.1996	0.0308	−0.0263	0.062*	
C3	−0.2497 (2)	0.10127 (19)	0.1282 (2)	0.0494 (6)	
H3	−0.3397	0.0855	0.1313	0.059*	
C4	−0.2034 (2)	0.20274 (17)	0.3231 (2)	0.0397 (5)	
H4A	−0.3006	0.1994	0.3237	0.048*	
H4B	−0.1782	0.2681	0.3225	0.048*	
C5	−0.1295 (2)	0.15704 (15)	0.4414 (2)	0.0330 (5)	
C6	−0.1955 (2)	0.10294 (17)	0.5200 (2)	0.0434 (6)	
H6	−0.2892	0.0949	0.503	0.052*	
C7	−0.1202 (2)	0.06098 (17)	0.6244 (2)	0.0446 (6)	
H7	−0.1625	0.0235	0.6777	0.053*	
C8	0.0186 (2)	0.07517 (15)	0.6487 (2)	0.0371 (5)	
H8	0.071	0.0472	0.7183	0.044*	
C9	0.0783 (2)	0.13177 (14)	0.56789 (19)	0.0302 (4)	
C10	0.2274 (2)	0.15549 (16)	0.58221 (19)	0.0349 (5)	
H10A	0.2649	0.1262	0.5135	0.042*	
H10B	0.236	0.2225	0.5728	0.042*	
C11	0.4251 (2)	0.07940 (18)	0.7160 (3)	0.0478 (6)	
H11	0.4625	0.0485	0.653	0.057*	
C12	0.4785 (3)	0.08368 (19)	0.8407 (3)	0.0569 (7)	
H12	0.5584	0.056	0.8802	0.068*	
C13	0.3895 (2)	0.13761 (18)	0.8967 (2)	0.0472 (6)	
H13	0.4008	0.1526	0.9824	0.057*	
N6	0.3934 (2)	0.12375 (14)	0.2741 (2)	0.0463 (5)	
O1	0.46828 (19)	0.16474 (15)	0.21131 (19)	0.0656 (5)	
O2	0.2747 (2)	0.10663 (15)	0.2250 (3)	0.0822 (7)	
O3	0.4345 (3)	0.10089 (19)	0.3834 (2)	0.1001 (9)	

### Atomic displacement parameters (Å^2^)

**Table:**

	*U*^11^	*U*^22^	*U*^33^	*U*^12^	*U*^13^	*U*^23^
Ag1	0.03190 (10)	0.04830 (12)	0.04040 (12)	−0.00783 (7)	0.00483 (8)	0.00771 (8)
N1	0.0312 (10)	0.0513 (11)	0.0371 (11)	−0.0051 (8)	0.0042 (8)	−0.0017 (9)
N2	0.0272 (9)	0.0495 (11)	0.0323 (10)	0.0015 (8)	−0.0017 (8)	−0.0004 (8)
N3	0.0264 (9)	0.0379 (9)	0.0242 (9)	−0.0010 (7)	0.0022 (7)	−0.0021 (7)
N4	0.0288 (9)	0.0437 (10)	0.0286 (9)	0.0067 (7)	0.0004 (7)	−0.0031 (8)
N5	0.0343 (10)	0.0439 (10)	0.0273 (10)	0.0078 (8)	−0.0011 (8)	−0.0022 (8)
C1	0.0387 (13)	0.0558 (14)	0.0350 (13)	0.0032 (11)	0.0039 (10)	−0.0005 (11)
C2	0.0455 (15)	0.0637 (16)	0.0419 (15)	0.0022 (12)	−0.0052 (12)	−0.0141 (12)
C3	0.0292 (12)	0.0692 (17)	0.0460 (15)	−0.0055 (11)	−0.0058 (11)	−0.0096 (12)
C4	0.0275 (11)	0.0533 (13)	0.0368 (13)	0.0064 (9)	0.0005 (9)	−0.0047 (10)
C5	0.0266 (11)	0.0411 (12)	0.0309 (12)	−0.0005 (8)	0.0031 (9)	−0.0066 (9)
C6	0.0282 (11)	0.0580 (15)	0.0448 (14)	−0.0100 (10)	0.0077 (10)	−0.0079 (11)
C7	0.0483 (15)	0.0517 (14)	0.0354 (13)	−0.0156 (11)	0.0121 (11)	0.0005 (11)
C8	0.0428 (13)	0.0395 (12)	0.0284 (11)	−0.0035 (9)	0.0039 (10)	0.0006 (9)
C9	0.0308 (11)	0.0341 (10)	0.0254 (10)	0.0001 (8)	0.0032 (8)	−0.0048 (8)
C10	0.0291 (11)	0.0501 (13)	0.0246 (11)	0.0002 (9)	0.0011 (9)	−0.0001 (9)
C11	0.0329 (12)	0.0548 (15)	0.0540 (16)	0.0134 (11)	0.0018 (11)	−0.0095 (12)
C12	0.0395 (14)	0.0646 (17)	0.0591 (18)	0.0201 (12)	−0.0158 (13)	−0.0025 (13)
C13	0.0441 (14)	0.0576 (15)	0.0340 (13)	0.0079 (11)	−0.0125 (10)	−0.0022 (11)
N6	0.0419 (12)	0.0504 (12)	0.0502 (13)	0.0160 (9)	0.0181 (11)	0.0031 (10)
O1	0.0448 (11)	0.0948 (15)	0.0613 (13)	−0.0115 (10)	0.0212 (10)	0.0002 (11)
O2	0.0391 (11)	0.0674 (14)	0.141 (2)	−0.0078 (10)	0.0174 (13)	−0.0111 (13)
O3	0.138 (2)	0.119 (2)	0.0488 (13)	0.0729 (18)	0.0331 (14)	0.0239 (13)

### Geometric parameters (Å, º)

**Table:**

Ag1—N5^i^	2.1652 (17)	C4—H4A	0.97
Ag1—N1	2.2772 (19)	C4—H4B	0.97
Ag1—N3	2.3126 (16)	C5—C6	1.382 (3)
N1—C1	1.325 (3)	C6—C7	1.381 (3)
N1—N2	1.348 (3)	C6—H6	0.93
N2—C3	1.338 (3)	C7—C8	1.382 (3)
N2—C4	1.461 (3)	C7—H7	0.93
N3—C9	1.338 (3)	C8—C9	1.384 (3)
N3—C5	1.347 (2)	C8—H8	0.93
N4—C11	1.340 (3)	C9—C10	1.508 (3)
N4—N5	1.356 (2)	C10—H10A	0.97
N4—C10	1.446 (3)	C10—H10B	0.97
N5—C13	1.331 (3)	C11—C12	1.358 (4)
N5—Ag1^ii^	2.1652 (17)	C11—H11	0.93
C1—C2	1.384 (3)	C12—C13	1.380 (4)
C1—H1	0.93	C12—H12	0.93
C2—C3	1.359 (4)	C13—H13	0.93
C2—H2	0.93	N6—O3	1.222 (3)
C3—H3	0.93	N6—O1	1.227 (3)
C4—C5	1.510 (3)	N6—O2	1.241 (3)
			
N5^i^—Ag1—N1	139.15 (7)	N3—C5—C6	121.5 (2)
N5^i^—Ag1—N3	137.05 (6)	N3—C5—C4	116.07 (18)
N1—Ag1—N3	83.34 (6)	C6—C5—C4	122.41 (19)
C1—N1—N2	105.18 (18)	C7—C6—C5	119.0 (2)
C1—N1—Ag1	134.79 (16)	C7—C6—H6	120.5
N2—N1—Ag1	118.94 (14)	C5—C6—H6	120.5
C3—N2—N1	111.20 (19)	C6—C7—C8	119.4 (2)
C3—N2—C4	128.7 (2)	C6—C7—H7	120.3
N1—N2—C4	120.01 (18)	C8—C7—H7	120.3
C9—N3—C5	119.42 (17)	C7—C8—C9	118.8 (2)
C9—N3—Ag1	118.76 (13)	C7—C8—H8	120.6
C5—N3—Ag1	120.62 (13)	C9—C8—H8	120.6
C11—N4—N5	111.05 (18)	N3—C9—C8	121.82 (19)
C11—N4—C10	127.36 (19)	N3—C9—C10	112.75 (18)
N5—N4—C10	120.55 (17)	C8—C9—C10	125.43 (19)
C13—N5—N4	105.05 (18)	N4—C10—C9	115.87 (18)
C13—N5—Ag1^ii^	134.18 (16)	N4—C10—H10A	108.3
N4—N5—Ag1^ii^	120.26 (13)	C9—C10—H10A	108.3
N1—C1—C2	111.0 (2)	N4—C10—H10B	108.3
N1—C1—H1	124.5	C9—C10—H10B	108.3
C2—C1—H1	124.5	H10A—C10—H10B	107.4
C3—C2—C1	105.2 (2)	N4—C11—C12	107.3 (2)
C3—C2—H2	127.4	N4—C11—H11	126.3
C1—C2—H2	127.4	C12—C11—H11	126.3
N2—C3—C2	107.4 (2)	C11—C12—C13	105.7 (2)
N2—C3—H3	126.3	C11—C12—H12	127.1
C2—C3—H3	126.3	C13—C12—H12	127.1
N2—C4—C5	111.67 (18)	N5—C13—C12	110.8 (2)
N2—C4—H4A	109.3	N5—C13—H13	124.6
C5—C4—H4A	109.3	C12—C13—H13	124.6
N2—C4—H4B	109.3	O3—N6—O1	120.6 (3)
C5—C4—H4B	109.3	O3—N6—O2	120.5 (3)
H4A—C4—H4B	107.9	O1—N6—O2	118.9 (2)

Symmetry codes: (i) *x*, −*y*+1/2, *z*−1/2; (ii) *x*, −*y*+1/2, *z*+1/2.

### Hydrogen-bond geometry (Å, º)

**Table:**

*D*—H···*A*	*D*—H	H···*A*	*D*···*A*	*D*—H···*A*
C3—H3···O1^iii^	0.93	2.49	3.206 (3)	134
C4—H4*A*···O1^iii^	0.97	2.48	3.347 (3)	149
C10—H10*A*···O3	0.97	2.38	3.277 (3)	154
C11—H11···O3^iv^	0.93	2.43	3.195 (3)	140
C13—H13···O1^v^	0.93	2.44	3.355 (3)	167

Symmetry codes: (iii) *x*−1, *y*, *z*; (iv) −*x*+1, −*y*, −*z*+1; (v) *x*, *y*, *z*+1.
