# Supplementary figures and images for: Crystal structure of catena-poly[[silver(I)-{μ-2,6-bis­[(1H-pyrazol-1-yl)meth­yl]pyridine-κ3 N 1,N 2:N 2′}] nitrate]
Source: Acta Crystallogr E Crystallogr Commun. 2015 Mar 7;71(Pt 4):m79–80. doi: 10.1107/S2056989015004120 (PMC4438834; doi:10.1107/S2056989015004120)

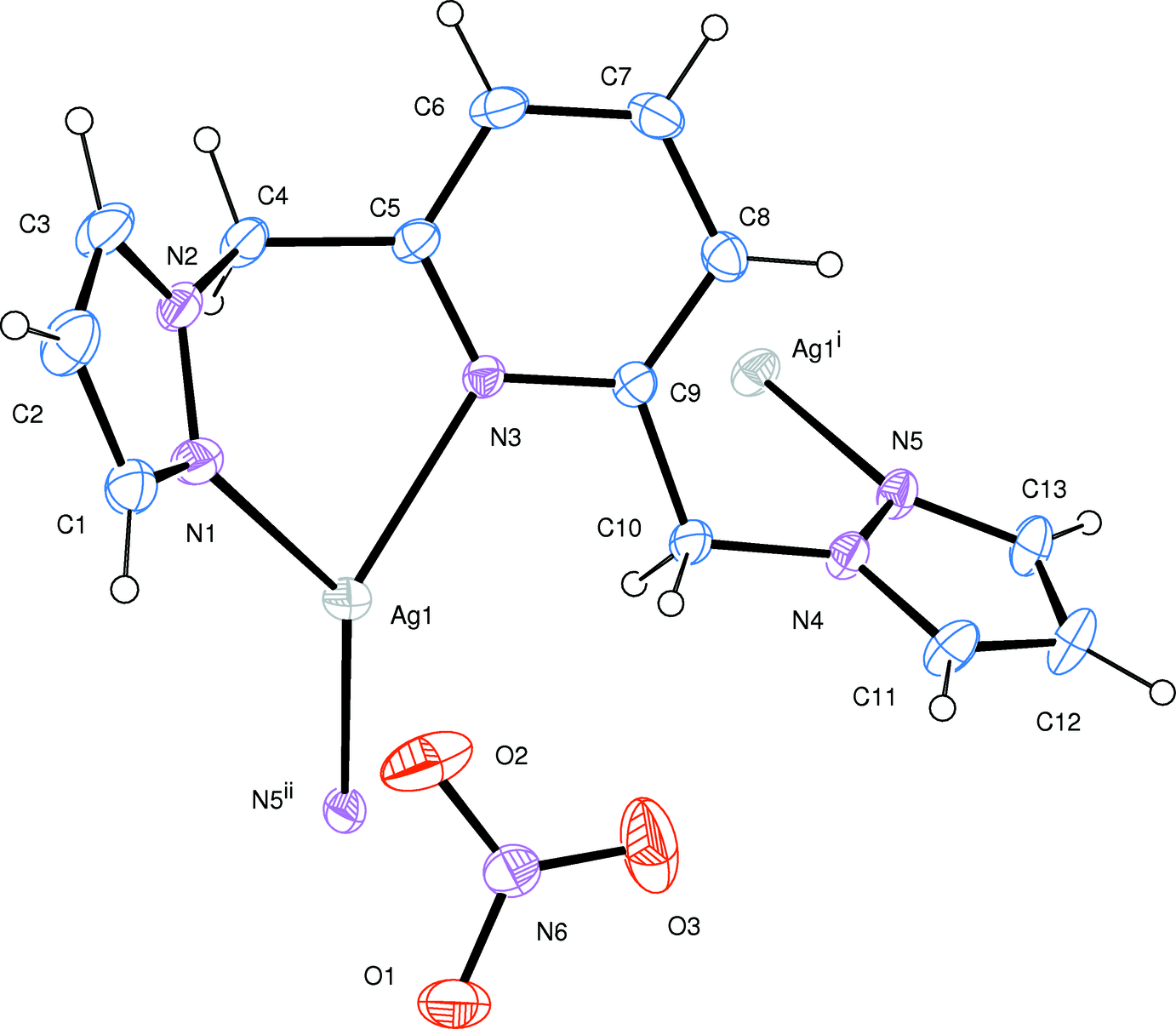

Supplement: Supplementary file 3 [file e-71-00m79-fig1.tif]

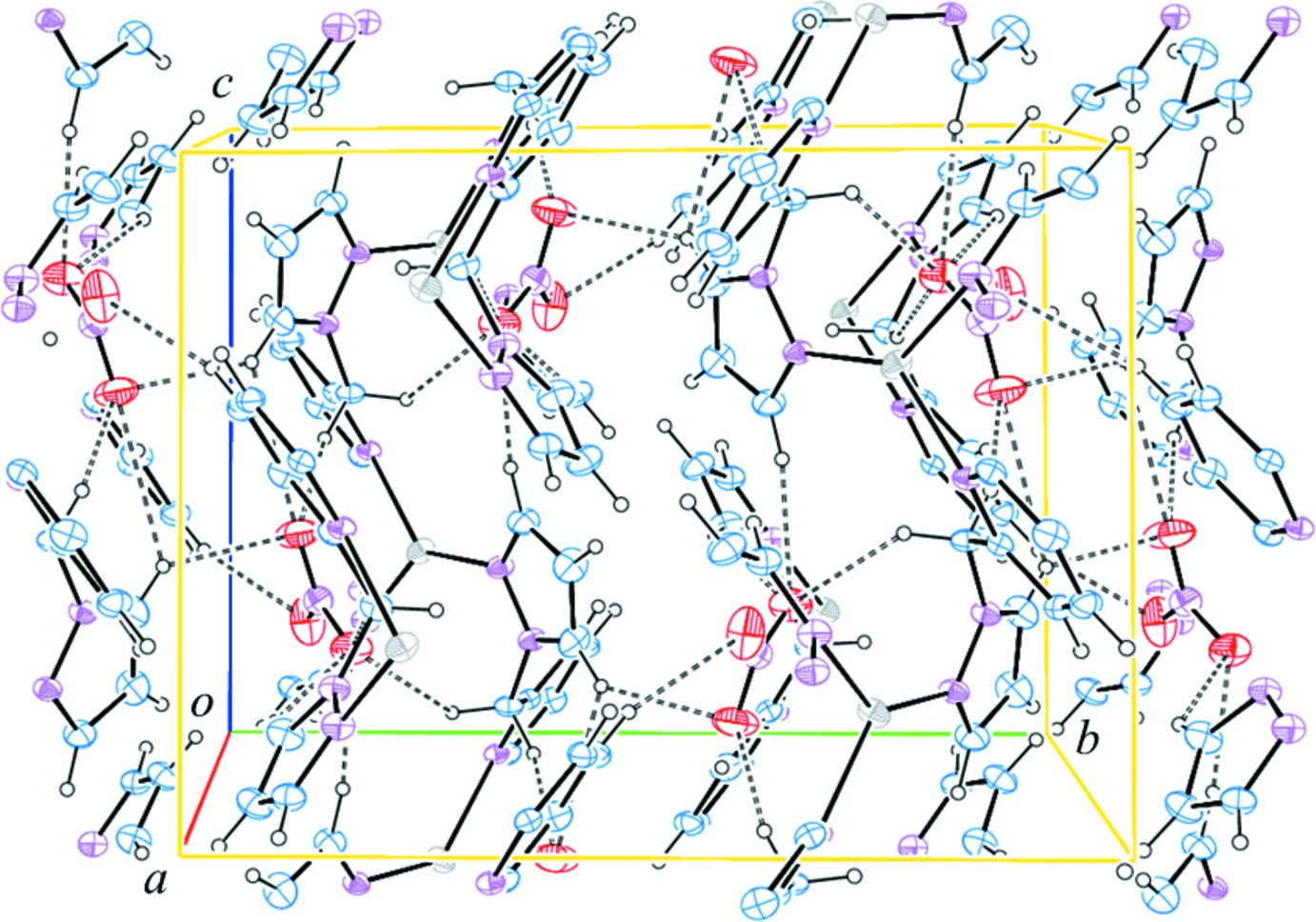

Supplement: Supplementary file 4 [file e-71-00m79-fig2.tif]
